# *In silico* analyses of deleterious missense SNPs of human apolipoprotein E3

**DOI:** 10.1038/s41598-017-01737-w

**Published:** 2017-05-30

**Authors:** Allan S. Pires, William F. Porto, Octavio L. Franco, Sérgio A. Alencar

**Affiliations:** 1 Programa de Pós-Graduação em Ciências Genômicas e Biotecnologia, Universidade Católica de Brasília, Brasília-DF, Brazil; 2 Centro de Análises Proteômicas e Bioquímicas, Pós-Graduação em Ciências Genômicas e Biotecnologia, Universidade Católica de Brasília, Brasília-DF, Brazil; 3 Porto Reports, Brasília-DF, Brazil; 4 S-Inova Biotech, Pós-graduação em Biotecnologia, Universidade Católica Dom Bosco, Campo Grande, MS Brazil

## Abstract

ApoE3 is the major chylomicron apolipoprotein, binding in a specific liver peripheral cell receptor, allowing transport and normal catabolism of triglyceride-rich lipoprotein constituents. Point mutations in ApoE3 have been associated with Alzheimer’s disease, type III hyperlipoproteinemia, atherosclerosis, telomere shortening and impaired cognitive function. Here, we evaluate the impact of missense SNPs in *APOE* retrieved from dbSNP through 16 computational prediction tools, and further evaluate the structural impact of convergent deleterious changes using 100 ns molecular dynamics simulations. We have found structural changes in four analyzed variants (Pro102Arg, Arg132Ser, Arg176Cys and Trp294Cys), two of them (Pro102Arg and Arg176Cys) being previously associated with human diseases. In all cases, except for Trp294Cys, there was a loss in the number of hydrogen bonds between CT and NT domains that could result in their detachment. In conclusion, data presented here could increase the knowledge of ApoE3 activity and be a starting point for the study of the impact of variations on *APOE* gene.

## Introduction

Apolipoproteins (Apo) compose a family of proteins involved in lipid metabolism, participating in many transport pathways, with major physiological importance. In humans, a large number of apolipoproteins that perform different functions have been described, including ApoA^1, 2^, ApoB^3, 4^, ApoC^5^, ApoD^6^, and ApoE^7, 8^. ApoA and ApoD have been described as components of the High Density Lipoprotein (HDL) transport, ApoA being the major component in plasma^2, 5^, whereas ApoB plays a critical role in the low-density lipoprotein (LDL) transport system^3, 4^. Meanwhile, ApoC has been described as a component of very low-density lipoprotein (VLDL)^5^; and ApoE is the major apoliprotein of chylomicrons.

ApoE is capable of binding to a specific liver peripheral cell receptor, allowing transport and normal catabolism of triglyceride-rich lipoprotein constituents^7, 8^. It is known that ApoE forms oligomers^9^ and, when bound to heparan sulfate proteoglycans (HSPG) and lipids, it adopts an active conformation that allows binding and transport of the low-density lipoprotein receptor (LDLR)^10–12^. Currently, three common isoforms of ApoE are known. These isoforms may be generated by polymorphisms in two different positions within coding regions of the *APOE* gene that lead to amino acid residue changes in positions 130 (site A) and 176 (site B) of the mature ApoE protein: ApoE2 (C130/C176), ApoE3 (C130/R176) and ApoE4 (R130/R176)^9, 11, 13^. As a result, these differences alter the ApoE function^14, 15^. ApoE isoforms have been associated with several human disorders, such as Alzheimer’s disease^16, 17^, type III hyperlipoproteinemia^12, 18^, atherosclerosis^19^, telomere shortening^20^, impaired cognitive function^21^ and infectious diseases^22, 23^. Some of these disorders could be associated with specific isoforms, such as type III hyperlipoproteinemia and Alzheimer’s disease, which are associated with ApoE2 and ApoE4^2, 16^, respectively.

In humans, the most common isoform is ApoE3, characterized as the wild type^12^, and this is the unique isoform with a fully elucidated structure, while the other isoforms have only partial structures (e.g. receptor binding domain). Since ApoE3 forms oligomers, some variations (F257A/W264R/V269A/L279Q/V287E) were needed to be inserted in the C-terminus to allow structure elucidation, making a monomeric ApoE3^9^. The ApoE3 structure can be divided into three structural domains: (i) the NT domain comprises the region between residues 1 and 167, (ii) the hinge domain from residues 168 to 205, and (iii) the CT domain from residues 206 to 299^9^. It is known that the CT domain undergoes structural changes when ApoE binds to lipids, leading to activation of the molecule^9, 12, 15^. Nevertheless, residues 140-160 from the NT domain are important for the interaction with LDL^24, 25^. Furthermore, since the protein is mostly stabilized by hydrogen bond interactions and salt bridges, loss of interactions of this type can cause folding errors or loss of affinity for ligands and they could be involved in disease development^9, 26, 27^. These interactions are very important for the correct folding of CT and Hinge domains^9^. In addition, the interaction between NT and CT exposes hydrophobic residues in CT, increasing lipid affinity^9^.

Several studies have shown the effect of point mutations on the functionality of ApoE3. When examining patients with lipoprotein glomerulopathy, Oikawa *et al*. (1991) found that the Arg163Pro point mutation could cause a lower affinity for the LDL receptor (LDLR)^28^. Also, Suehiro *et al*. (1990) demonstrated that the substitution of the same arginine at position 163 of mature protein by a histidine might lead to a lower receptor interaction, increasing the risk for dysbetalipoproteinemia^25, 29^. However, despite the fact that several point mutations present in the coding region of *APOE* have been suggested to be associated with human diseases, the potential impact of missense SNPs described in the dbSNP database has not yet been evaluated.

Currently, computational methods designed to predict the impact of amino acid residue changes in proteins have been widely used in order to assess whether changes are deleterious or not^30^. Among several existing tools, four different groups can be defined based on their methodology: protein-sequence and structure, sequence homology, supervised-learning, and consensus methods^31^.

Although there are currently a number of tools used to predict the potential structural and functional impact caused by amino acid changes, these tools are not highly accurate^32^. However, they can still be used as an initial filter of potentially deleterious changes^31^. Then, more refined analysis, such as molecular dynamics simulations, can be used in order to evaluate more precisely the structural impact caused by amino acid changes^31, 33^.

The use of molecular dynamics simulations enables the evaluation of structural changes in molecules over a short time window, also allowing observations of changes in physicochemical properties and interactions in simulated environments^34^. However, the use of this method requires high computational power, making it difficult to simulate longer periods. Hence, simulations are limited to just hundreds of nanoseconds. Nevertheless, this method has been widely used to evaluate changes in protein structure caused by point mutations and missense SNPs, such as in the study of α- and β-defensins^35^, p53^36^, lamin A/C protein^37^, guanylin^31^, aldosterone synthase^38^ and aurora-A kinase^33^.

Here, we evaluate the impact of *APOE* missense SNPs from dbSNP by means of a number of computational prediction tools, and further evaluate the structural impact of potentially deleterious changes using molecular dynamics simulations. Our hypothesis is that these variations could cause a significant impact on the protein structure and stability.

## Material and Methods

### Datasets

The dbSNP database contains SNPs and multiple small-scale variations that include insertions/deletions, microsatellites, and non-polymorphic variants^39^. Using the dbSNP search engine available from the NCBI, only human validated *APOE* SNPs and non-polymorphic single nucleotide variants (SNVs) were filtered. The ApoE3 protein sequence (NCBI Accession: NP_000032.1) was retrieved from the NCBI Protein database (http://www.ncbi.nlm.nih.gov/protein), and the protein structure file of ApoE3 (PDB ID: 2L7B) was obtained from the RCSB Protein Data Bank^9, 40^. The frequency data of missense SNPs found in the *APOE* gene were obtained from the publicly available 1000 Genomes Project (phase I) (http://www.1000genomes.org)^41^. The variant format file (phase 1 release v3.20101123) corresponding to chromosome 19 contained the frequencies of all SNPs identified in the genomes of 1,092 individuals from 14 populations obtained through a combination of low-coverage (2–6x) whole-genome sequence data, targeted deep (50–100x) exome sequencing and dense SNP genotype data. The 14 populations studied were grouped by the predominant component of ancestry into four super-populations: African (AFR) (246 samples), East Asian (ASN) (286 samples), European (EUR) (379 samples) and Ad Mixed American (AMR) (181 samples).

### SNP Selection

As rare SNPs occur at very low frequencies (<1%), there is great concern to avoid confounding putative SNPs with sequencing errors common in next-generation sequencing technologies. Therefore, initially we selected from dbSNP only the ones that fit at least one of the following conditions: (i) it has been sequenced in the 1000 Genomes Project; (ii) it has frequency or genotype data (minor alleles observed in at least two chromosomes); and (iii) it has multiple, independent submissions to the refSNP cluster. Then, in order to evaluate the potential functional impact of the obtained *APOE* missense SNPs, we utilized a total of 16 prediction tools, divided into four different methods, as shown below. We filtered all missense SNPs that were classified as deleterious by at least three tools in each of the four groups, and denominated these as convergent deleterious predicted SNPs.

#### Sequence homology-based methods

The following methods based on sequence homology principles were used to produce missense SNP functional predictions: Sorting Intolerant From Tolerant (SIFT)^42^, Provean^43^, Mutation Assessor and Panther^44, 45^.

#### Supervised learning methods

Supervised learning algorithms used for missense SNP impact prediction included neural networks (SNAP)^46^, support vector machines (MutPred and SuSPect) and random forests (EFIN)^47–49^.

#### Protein sequence and structure-based methods

The following methods either combine information from protein sequence and structure or use protein structural information alone to analyze missense variants: PolyPhen^50^, Site Directed Mutator (SDM)^51^, Fold-X^52^ and PoPMuSiC^53^.

#### Consensus-based methods

In order to obtain a consensus score based on many different SNP impact prediction strategies, the following types of consensus software were used: Condel^54^, Meta-SNP^55^, PON-P2 and PredictSNP^56, 57^.

### Evolutionary Conservation Analysis

The ConSurf server is a tool for estimating the evolutionary conservation of amino acid positions in a protein molecule based on the phylogenetic relations between homologous sequences^58^. Using the ApoE3 protein sequence (NCBI Accession: NP_000032.1)^40, 59^, ConSurf, in ConSeq mode, a search was carried out for close homologous sequences using CSI-BLAST (3 iterations and 0.0001 e-value cutoff) against the UNIREF-90 protein database^60, 61^. The maximum number of homologs to collect was set as 150, and the minimal and maximal percentage ID between sequences were set as 35 and 95, respectively. The multiple sequence alignment and calculation methods were left as default (MAFFT-L-INS-i and Bayesian). The sequences were then clustered and highly similar sequences removed using CD-HIT^62^. Position-specific conservation scores were computed using the empirical Bayesian algorithm^63^.

### Signal Peptide Prediction

In order to verify the impact of convergent deleterious SNPs in the signal peptide, Phobius^64^ and SignalP 4.0^65^ were used for signal peptide topology prediction.

### Molecular Modeling

The structural models containing each missense SNP were separately made by means of MODELLER 9.14^66^ using the class automodel with default settings. The template used as wild type was the monomeric ApoE3 structure (PDB ID: 2L7B)^59^. One hundred models were generated for each variant. The best models were selected according to DOPE (Discrete Optimized Protein Structure) score, which indicates the most probable structure. The best models were evaluated by PROSA II^67^ and PROCHECK^68^ softwares. PROSA II evaluates the model quality while PROCHECK evaluates the stereochemical quality of the model through Ramachandran plot. Good quality models were selected by more than 90% of residues in most favoured and additional allowed regions. The visualization of the structures was done in PyMOL (http://www.pymol.org).

### Molecular dynamics simulation

The molecular dynamics simulations of the wild type and the four variant structures were performed by GROMACS 4 computational package using the GROMOS96 43A1 force field^69^. Structures are immersed in water cubic boxes with a 12 Å distance between the edge of the box and the protein. The simulations were done under ionic strength conditions (0,2 M NaCl)^70^. The box was filled using the Single Point Charge water model^71^. The dynamics used the wild type and variants three-dimensional models as initial structures. Additional chlorine ions were also inserted into the complexes with positive charges in order to neutralize the system charge. Geometry of water molecules was constrained by using the SETTLE algorithm^72^. Atomic connections were made through LINCS algorithm^73^. Electrostatic corrections were made by Particle Mesh Ewald algorithm^74^, with a threshold of 1.4 nm to minimize the computational time. The same cut-off radius was applied for van der Waals interactions. The steepest descent algorithm was applied to minimize system energy for 50,000 steps. After the energy minimization, the temperature (NVT ensemble) and pressure (NPT ensemble) systems were normalized to 300 K and 1 bar, respectively, each per 100 steps. The velocity-rescaling thermostat and the Parrinello-Rahman barostat were used for normalization of temperature and pressure, respectively. Full simulation of the system was made by 100 ns using the leap-frog algorithm as the integrator.

### Analyses of molecular dynamics trajectories

Molecular dynamics simulations were analyzed by means of the backbone root mean square deviation (RMSD), radius of gyration (Rg) and solvent accessible surface area (SASA) using the g_rms, g_gyrate and g_sas built in functions of the GROMACS package^69^, respectively. The essential dynamics was performed using the g_covar and g_anaeig utilities of the GROMACS package. The number of hydrogen bonds between the NT domain (residues 1–167) and the CT domain (residues 206–299) was analyzed using g_hbond, also from the GROMACS package. In addition, we analyzed the interactions between known regions of the protein previously described by Chen *et al*.^9^. Rg, SASA and the number of hydrogen bonds were plotted as boxplots, because these allow the visualization of the fluctuation and the range in which at least 50% of the data lies.

## Results

### Distribution and Frequency of *APOE* SNPs

**Figure 1:**
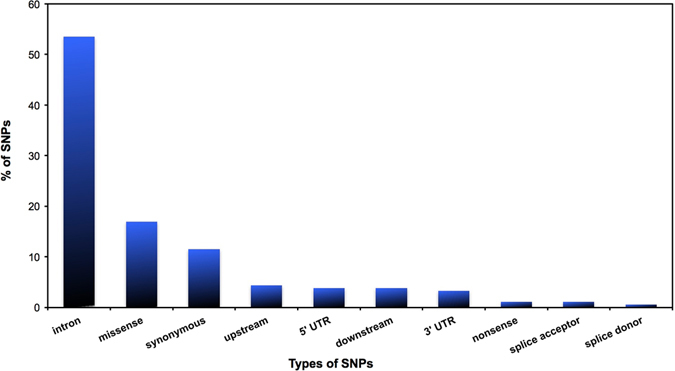
Distribution of SNPs within the APOE gene. The distribution was based on amino acid coding regions (missense, synonymous, nonsense, splice acceptor and splice donor) and on non-coding regions (intronic, upstream and downstream). It can be seen that the majority of the SNPs occur in non-coding regions: 53.6% in introns, 4.4% in upstream regions, 3.8% in 5′ UTR, 3.8% downstream and 3.3% in 3′ UTR. In the coding regions, the majority of the SNPs are missense (16.9%), followed by synonymous (11.5%), nonsense (1.1%), splice acceptor (1.1%) and splice donor (0.6%) variants.

Out of 183 validated *APOE* SNPs, 31 are missense, 21 are synonymous, and two are nonsense variants. There are also 98 intronic, 7 5′ UTR, 6 3′ UTR, 7 downstream, 8 upstream, 1 splice donor and 2 splice acceptor variants. A graphical representation of the distribution of SNPs in the coding and non-coding regions of the gene represented in terms of percentage is shown in Fig. 1. Frequency information was obtained from the 1000 Genomes Project for eight *APOE* missense SNPs (Table S1). All SNPs retrieved from the 1000 Genomes Project are disposed on Table S1. Five of them are rare SNPs with Global Allele Frequency (GAF) values below 1% and occurring only in one of the four populations studied, while the other three variants (Cys130Arg, Arg163Cys and Arg176Cys) have GAFs ≥1%. These variants represent ApoE4 (Cys130Arg), ApoE2* (Arg163Cys) and ApoE2 (Arg176Cys).

### ApoE3 Convergent Deleterious Predicted SNPs

**Figure 2:**
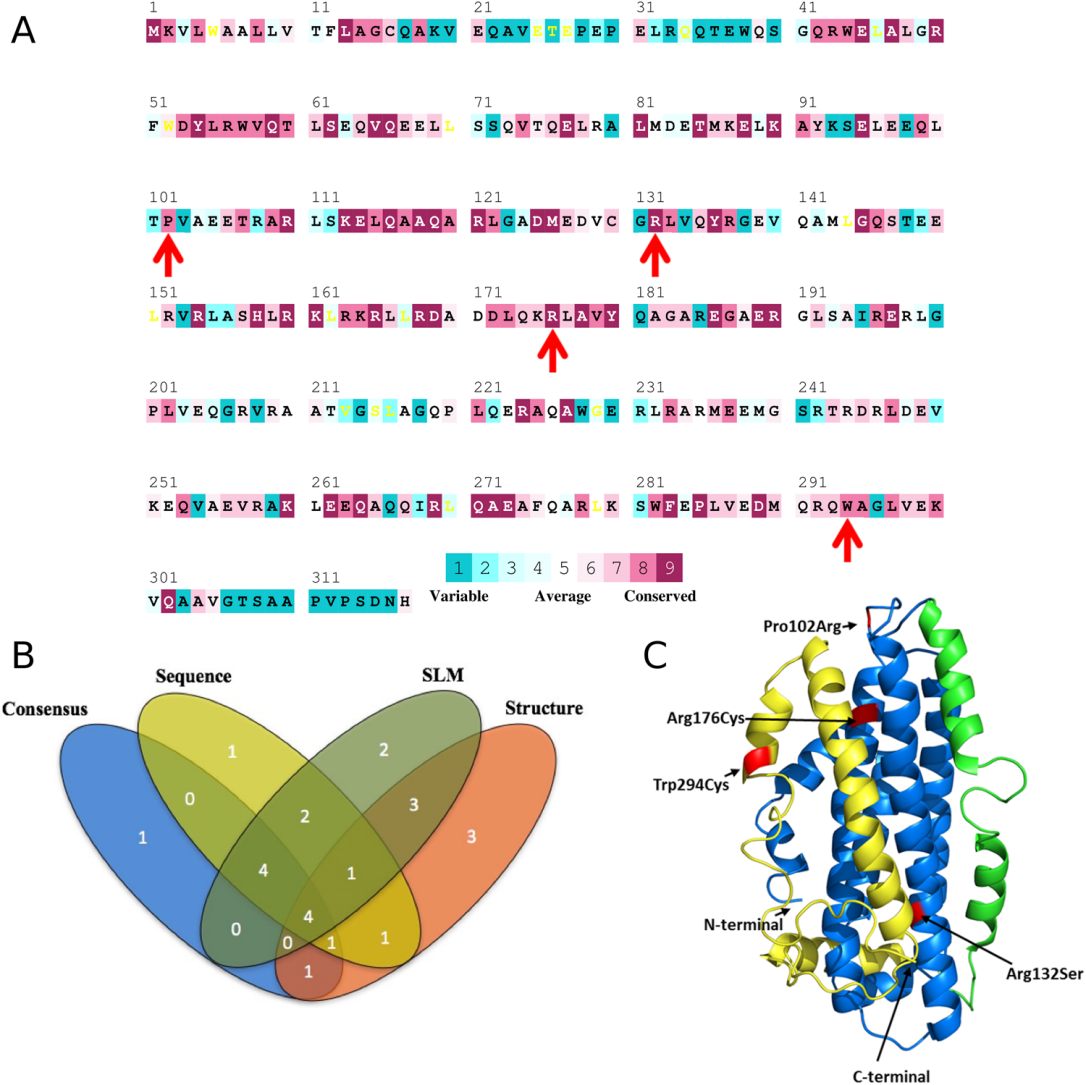
Missense SNPs identified in the APOE gene and Structural domains of native ApoE3. Conservation pattern of amino acid residues within the mature peptide region of ApoE3 obtained from multiple sequence alignment using ConSurf. Color intensity increases with degree of conservation. The amino acids are coloured based on their conservation grades and conservation levels. A grade of 1 indicates rapidly evolving (variable) sites, which are colour-coded in turquoise; 5 indicates sites that are evolving at an average rate, which are coloured white; and 9 indicates slowly evolving (evolutionarily conserved) sites, which are colour-coded in maroon. The four convergent deleterious predicted SNPs are marked below the peptide sequence as red arrows (**A**). Venn diagram showing the relationships between missense SNPs predicted as deleterious by the four different groups (sequence homology, supervised-learning (SLM), protein-sequence and structure, and consensus-based methods). A total of four convergent deleterious predicted SNPs (classified as deleterious by at least three tools in each of the four different groups) were obtained (**B**). Structural domains of native ApoE3. In blue is represented the NT domain, CT domain is represented in yellow and hinge region is showed in green. In red are highlighted the different variations analyzed in this work, identified by arrows (**C**).

There are currently a wide variety of computational tools used for predicting the effects of missense SNPs on protein function. In general, depending on the strategy, these tools can be classified into four groups: sequence homology, supervised-learning, protein-sequence and structure, and consensus-based methods. We filtered all missense SNPs that were classified as deleterious by at least three tools in each of the four groups (Table S2). A total of four SNPs (Pro102 Arg, Arg132Ser, Arg176Cys and Trp294Cys), which we previously named as convergent deleterious predicted SNPs^31^ were obtained from this filtration (Table 1). Only three SNPs (Thr11Ala, Ala14Thr and Ala18Thr) occur within the signal peptide region, and all remaining SNPs occur within the mature peptide region (Fig. 2A).

**Table 1:** Results of *APOE* convergent deleterious predicted SNPs analyzed by 16 prediction tools classified in four different groups.

SNP rs ^#^	Amino Acid Change^a^	ValidationMethod^b^	Sequence-Based^c^				SLM-Based^c^				Consensus-Based^c^				Structure-Based^c^			
			SIFT	Provean	Mutation Assessor	Panther	MutPred	EFIN	SNAP	SuSPect	Condel	MetaSNP	PON-P2	Predict SNP	PolyPhen	SDM	Fold-X	PoPMuSiC
rs11083750:C > A	Pro102Arg	Cluster	D	D	D	U	N	D	D	D	D	D	P	D	D	N	DT	DT
rs11542041:C > A	Arg132Ser	1000 G	D	D	D	D	D	D	D	D	N	D	P	D	D	D	DT	DT
rs7412:C > T	Arg176Cys	1000 G, cluster, freq.	D	D	D	D	D	D	D	D	D	D	N	D	D	N	DT	DT
rs557715042:G > T	Trp294Cys	1000 G, freq.	D	D	D	U	D	D	D	N	D	N	P	D	D	D	DT	DT

^a^APOE amino acid positions is relative to GenBank Accession number NP_000032.1.

^b^1000G: SNP has been sequenced in the 1000 Genomes Project; freq.: Validated by frequency or genotype data: minor alleles observed in at least two chromosomes; cluster: Validated by multiple, independent submissions to the refSNP cluster.

^c^N: Neutral; D: Deleterious; ST: Stabilizing; DT: Destabilizing; P: Pathogenic; U: Unknown.

In addition, we analyzed the evolutionary conservation of all missense SNPs within the mature region of ApoE3 using ConSurf^58, 75^. ConSurf exploits evolutionary variation in multiple sequence alignments in order to determine the degrees of conservation. The results from this analysis showed that the majority of the variations (66.7%) occur in sites classified as “conserved” (Fig. 2A), including all four convergent deleterious predicted SNPs (Pro102Arg, Arg132Ser, Arg176Cys and Trp294Cys).

### The Thr11Ala, Ala14Thr and Ala18Thr Variants Seem not to Alter the Signal Peptide

In order to evaluate the impact of Thr11Ala, Ala14Thr and Ala18Thr in the signal peptide, two prediction servers were used (Phobius and SignalP 4.0). However, none of them indicated any changes in the signal peptide topology.

### The impact of variations on protein structure

**Figure 3:**
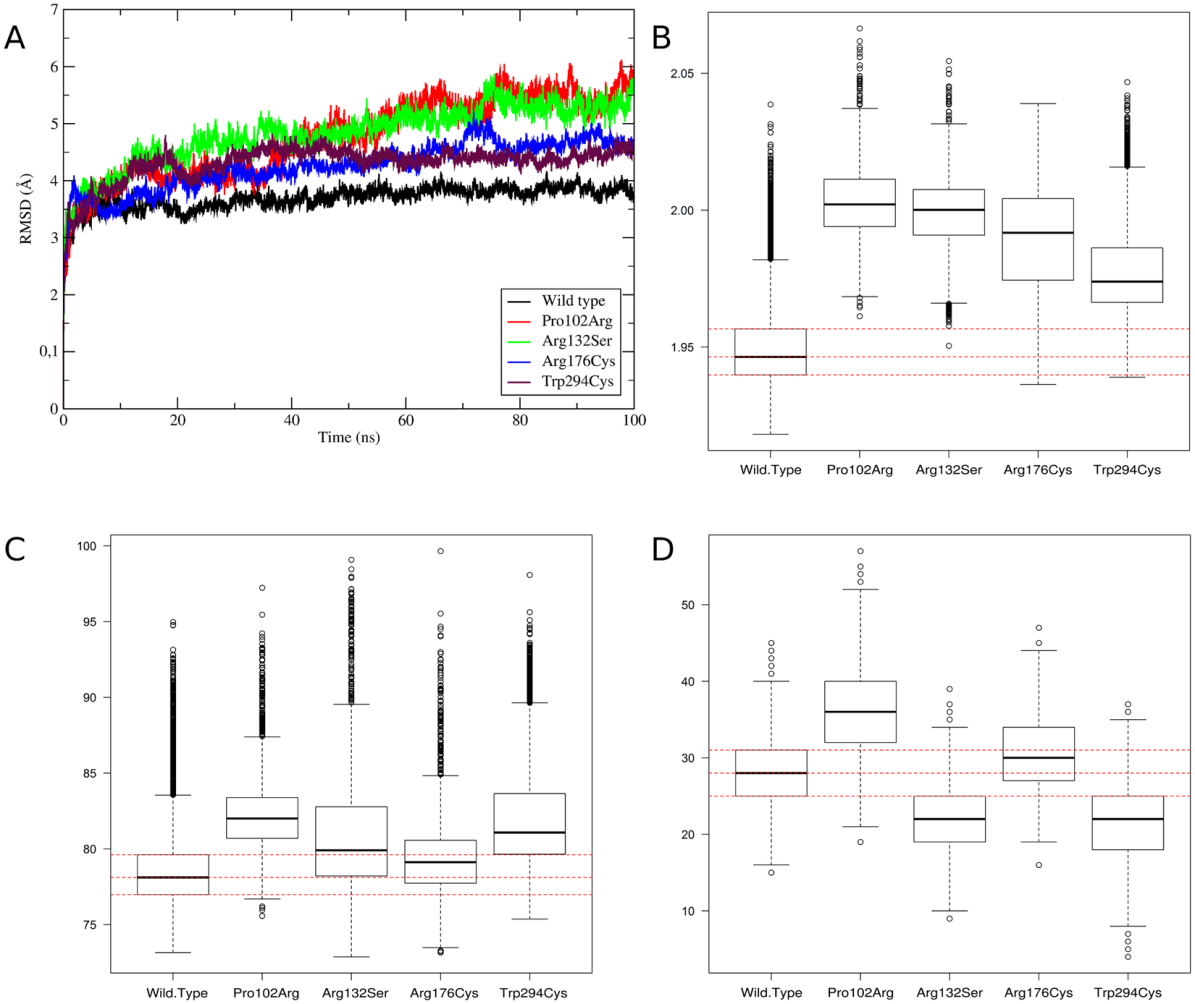
ApoE3 native and variants trajectories analyses. In Backbone RMSD variation the variants are identified in the plots by different colors (**A**). Radius of gyration (**B**), solvent accessible surface area (**C**) and number of hydrogen bonds (**D**) are plotted in boxplots. On backbone RMSD (**A**) the variants are identified in the plots by colors: Native structure (black), Pro102Arg (red), Arg132Ser (green), Arg176Cys (blue) and Trp296Cys (turquoise). Only the number of hydrogen bonds between NT and CT domains was computed (**D**). Dotted red lines on solvent accessible surface area, radius of gyration and number of hydrogen bonds plots indicate the reference values of wild type. The solvent accessible surface and radius of gyration values are in nm² and to RMSD values in Å.

ApoE3 native structure is characterized by ten α-helices stabilized by hydrogen bonds, salt bridges and hydrophobic interactions (Fig. 2C). The monomeric ApoE3 (PDB ID: 2L7B) was used to construct the variant structures. Since the ApoE3 monomeric structure has some modifications in the C-terminal, we modeled the native structure by the substitution of respective residues in the C-terminus. Table S3 summarizes the validation assessments. We performed molecular dynamics simulations to evaluate which probable structural changes occur within each modelled ApoE3 structure. The best model for each variant was simulated for 100 ns. The analysis of RMSD was carried out to measure differences in movement between native and variant backbones. The RMSD analysis showed that the native structure had little variation during the simulation time, ranging from 3 to 4 Å (Fig. 3A). Despite that, all analyzed variants presented a higher variation in the backbone of the protein ranging from 3 to 6 Å in Pro102Arg and Arg132Ser and from 3 to 5 Å in Arg176Cys and Trp294 simulations (Fig. 3A).

In contrast, analysis of the radius of gyration showed wide differences between variant and wild structures, with an increase for all variants (Fig. 3B). The protein flexibility was also analyzed, by means of essential dynamics, showing that all the variants had a gain in flexibility (Figure S1). Therefore, solvent accessible surface area and radius of gyration were measured in order to evaluate the maintenance of protein packing. The solvent accessible surface area analysis of the variant structures showed little difference between the wild type structure and Arg132Ser and Arg176Cys variants, with little or no increase (Fig. 3C). However, Pro102Arg and Trp294Cys showed a higher increase on this property (Fig. 3C).

Structural changes in CT may reduce the ApoE3 affinity to lipids^59^. Since NT stabilizes CT, we verified whether some of the convergent deleterious SNPs could affect the number of hydrogen bonds made between CT and NT amino acid residues. There were differences between wild type and variant structures in all cases. While the Arg132Ser and Trp294Cys variants showed a decrease in the number of hydrogen bonds in comparison to the wild type structure (Fig. 3D), the Pro102Arg variant exhibited an increase (Fig. 3D). Moreover, Arg176Cys showed a little increase in the number of hydrogen bonds in comparison to the wild type structure, however, almost the same behavior as the wild type (Fig. 3D). Furthermore, we analyzed differences in the number of interactions between known structural regions in native and variant structures over time. From this, we measured the variant effects on known interactions of native structure. In analyzes with NT and CT domains, almost all variants presented differences when compared to the native structure, with a decrease in Arg132Ser and Trp294Cys variants, an increase in Pro102Arg and the same number of interactions in Arg176Cys (Fig. 3D). However, only Trp294Cys presented a loss of hydrogen bonds between known regions in ApoE3 (Figure S2 and Table S4). Meanwhile, the other three variants presented a great increase in these interactions. However, Pro102Ser presented the greatest impact on the number of hydrogen bonds between the structural domains, with an average gain of about 17 hydrogen bonds in relation to the native structure.

## Discussion

ApoE3 presents an helical structure stabilized by hydrogen bonds and salt bridges^59^. This characteristic confers protein plasticity and capacity of large conformational changes, important for the activity performed by this protein. Here, we used molecular dynamics simulations to assess conformational changes caused by the presence of missense SNPs that lead to amino acid residue changes in the coded protein. We were able to simulate a protein of 299 amino acid residues for 100 ns. For short peptides, it is not difficult to reach this simulation time^31^, but for proteins greater than 200 amino acids it is common to simulate for less than 10 ns^36–38^, with few exceptions being simulated for more than 100 ns^33^.

All of the four variants analyzed here are present in conserved regions of the protein (Fig. 2B). Therefore, the implementation of 16 prediction tools to pre-filter potentially damaging SNPs present in the *APOE* gene could in fact lead to the discovery of variations that have an impact on protein structure and, consequently, on its function. Furthermore, the use of a consensus of different types of tools (e.g sequence homology-based, supervised learning method and protein sequence and structure-based) to screen potentially damaging SNPs increases their prediction accuracy. Out of the four variants, Pro102Arg presented an increase in all analyses compared to the native protein (Fig. 3D). Interestingly, despite a gain in the number of hydrogen bonds between both CT and NT, as well as between known structural domains, this variant presents the largest differences relative to the wild type structure (Fig. 3).

However, this variant has not been associated with any diseases reported in the literature yet. It is known that ApoE4 is associated with hyperlipidemia^2^, nevertheless, the double mutant (Cys112Arg/Pro102Arg) has not been described as having this association^76, 77^. Despite the compensatory effect of Pro102Arg on ApoE4, in ApoE3 it could be deleterious due to the gain in radius of gyration, surface and hydrogen bonds.

On the other hand, the Trp294Cys and Arg132Ser variants presented loss in hydrogen bonds between CT and NT domains (Fig. 3D). This occurs due to the loss of a hydrophobic amino acid in the CT domain. Besides that, the substitution of Trp294 could interfere with lipid interaction mediated by the CT domain, causing loss of affinity^59^. This step of interaction with lipid was previously associated with activation of the protein, starting the essential structural changes that expose the LDLR binding region in the NT domain^59^. Moreover, previously, single point changes in CT were used to inhibit the oligomerization of ApoE3^59^. Therefore, it is possible that missense SNPs present in this region could also interpose the normal behavior of the protein. On the other hand, Arg132Ser is important in interdomain interaction, performing two hydrogen bonds with CT domain residues (Gln235 and Glu238)^59^. Then, loss of hydrogen bonds caused by Arg132Ser could generate the separation of the CT and NT domains, exposing the LDLR interaction domain without the activation by lipids^59^.

Finally, the Arg176Cys variant presented a more similar behavior compared to the native protein (Fig. 3). Despite this, given the large variation in the radius of gyration analysis of the Arg176Ser variant and the increase of the RMSD, it is possible that this variant generates an opening and closing movement of the Arg176Ser variant, which causes the highest variation on radius of gyration. The Arg176Cys variant characterizes the E2 isoform, which is associated with diseases such as hyperlipoproteinemia III^78, 79^ and atherosclerosis^78^. Our analysis showed that this variant could result in a change in affinity between ApoE and LDLR, generating the clinical condition^13, 24, 59, 80^. The Arg176Cys variant did not show great differences in number of hydrogen bonds (Fig. 3D) or solvent accessible surface analyses (Fig. 3C). Furthermore, this variation has a GAF ≥1%, being the most common variation in this study.

## Conclusions

Although many variations have been identified in the *APOE* gene, the potential structural and functional impact of many of them have not been analyzed yet. However, the four analyzed variants could lead the protein to lose affinity with lipids. The loss of hydrogen bonds between NT and CT domains viewed in variants may be an important factor for research into association between diseases and ApoE variations. Furthermore, the similarity in ApoE2 and other variations could be significant to analyses of impact of these variations and their association with diseases. In conclusion, data presented here could increase the knowledge of ApoE3 activity and be a starting point for the study of impact of variations on the *APOE* gene.

## Electronic supplementary material

Supplementary Information
