# Supplementary material for: In silico analyses of deleterious missense SNPs of human apolipoprotein E3
Source: Sci Rep. 2017 May 30;7:2509. doi: 10.1038/s41598-017-01737-w (PMC5449402; doi:10.1038/s41598-017-01737-w)
Supplement: Supplementary file 1 — Supplementary Information [file 41598_2017_1737_MOESM1_ESM.docx]

***In silico* analyses of deleterious missense SNPs of human apolipoprotein E3**

Allan S. Pires^1,2^, William F. Porto^1,2^, Octavio L. Franco^1,2,3^, Sergio A. Alencar^1*^

^1^Programa de Pós-Graduação em Ciências Genômicas e Biotecnologia, Universidade Católica de Brasília, Brasília-DF, Brazil.

^2^Centro de Análises Proteômicas e Bioquímicas, Pós-Graduação em Ciências Genômicas e Biotecnologia, Universidade Católica de Brasília, Brasília-DF, Brazil.

^3^S-Inova Biotech, Pós-graduação em Biotecnologia, Universidade Católica Dom Bosco, Campo Grande, MS, Brazil.

^*^ Corresponding author

E-mail: sergiodealencar@gmail.com (SA)

#

# Supplementary Information

**Table S1 – Allele frequencies of *APOE* missense SNPs obtained from the 1000 Genomes Project.**

| **SNP rs #** | **Amino Acid** | **Global Allele** | **Global Allele** | **RelativeAlleleFrequency (%)^c^** | | | |
| --- | --- | --- | --- | --- | --- | --- | --- |
|  | **Change^a^** | **Count** | **Frequency (%)^b^** | **ASN** | **AMR** | **AFR** | **EUR** |
| rs121918392:G>A | Glu21Lys | 1 | 0.05 | 0.17 | - | - | - |
| rs201672011:G>A | Glu31Lys | 1 | 0.05 | - | 0.28 | - | - |
| rs769452:T>C | Leu46Pro | 3 | 0.14 | - | - | - | 0.4 |
| rs429358:T>C | Cys130Arg | 326 | 15.0 | 9.0 | 11.0 | 26.0 | 14.0 |
| rs769455:C>T | Arg163Cys | 21 | 1.0 | - | 1.0 | 4.0 | - |
| **rs7412:C>T** | **Arg176Cys** | 162 | 7.0 | 8.0 | 6.0 | 9.0 | 7.0 |
| rs140808909:G>A | Glu262Lys | 2 | 0.09 | 0.35 | - | - | - |
| rs190853081:G>A | Glu263Lys | 2 | 0.09 | 0.35 | - | - | - |

Convergent deleterious SNPs are in bold face.

^a^APOE amino acid positions is relative to GenBank Accession number NP_000032.1.
^b^The global allele frequency was obtained by analyzing genotype data from 1092 individuals.
^c^The populations analyzed represent four super populations: African (AFR), Ad Mixed American (AMR), East Asian (ASN) and European (EUR). The relative allele frequencies were obtained from the allele frequencies of 246, 181, 286 and 379 individuals sequenced in the AFR, AMR, ASN and EUR super populations, respectively.

**Table S2 – Complete Prediction results of *APOE* missense SNPs analyzed by 16 prediction tools classified in four different groups.**

| **SNP rs #** | **Amino AcidChange^a^** | **ValidationMethod^b^** | **Sequence-Based^c^** | | | | **SLM-Based^c^** | | | | **Consensus-Based^c^** | | | | **Structure-Based^c^** | | | |
| --- | --- | --- | --- | --- | --- | --- | --- | --- | --- | --- | --- | --- | --- | --- | --- | --- | --- | --- |
|  |  |  | **SIFT** | **Provean** | **Mutation Assessor** | **Panther** | **MutPred** | **EFIN** | **SNAP** | **SuSPect** | **Condel** | **MetaSNP** | **PON-P2** | **Predict SNP** | **PolyPhen** | **SDM** | **Fold-X** | **PoPMuSiC** |
| rs144354013:G>A | Thr11Ala | 1000G | N | N | D | N | N | N | N | D | D | N | U | N | N | U | U | U |
| rs559532612:G>A | Ala14Thr | 1000G | N | N | N | N | N | N | N | D | N | N | U | N | N | D | U | U |
| rs533904656:G>A | Ala18Thr | 1000G, freq. | D | D | D | N | D | N | N | D | D | N | U | N | N | D | U | U |
| rs121918392:G>A | Glu21Lys | 1000G | N | N | D | N | D | D | D | D | D | N | U | N | D | N | ST | DT |
| rs201672011:G>A | Glu31Lys | 1000G, freq. | N | N | N | U | D | D | D | N | D | N | U | N | N | N | ST | ST |
| rs769452:T>C | Leu46Pro | 1000G, cluster, freq. | N | N | D | N | N | D | D | N | D | D | P | D | D | D | DT | DT |
| rs11542029:C>T | Arg50Cys | Cluster | D | D | D | D | D | N | D | D | D | D | P | D | D | N | ST | DT |
| rs370594287:G>C | Gln64His | 1000G, freq. | N | N | D | N | D | D | D | N | D | N | U | N | D | D | DT | ST |
| rs557845700:G>T | Met82Ile | 1000G | N | N | N | N | D | N | N | N | N | N | U | N | N | N | DT | DT |
| rs577618688:A>G | Gln99Arg | 1000G, freq. | D | N | D | N | N | N | D | D | D | N | P | D | D | N | ST | DT |
| rs11542040:C>A | Pro102Thr | Cluster | D | D | D | U | D | D | N | D | N | D | P | D | D | N | DT | ST |
| **rs11083750:C>A** | **Pro102Arg** | Cluster | D | D | D | U | N | D | D | D | D | D | P | D | D | N | DT | DT |
| rs429358:T>C | Cys130Arg | 1000G, cluster, freq. | N | N | N | N | D | N | N | N | D | N | U | N | N | D | DT | DT |
| **rs11542041:C>A** | **Arg132Ser** | 1000G | D | D | D | D | D | D | D | D | N | D | P | D | D | D | DT | DT |
| rs573658040:C>T | Arg137Cys | 1000G | D | N | D | D | D | N | D | D | D | D | P | D | D | N | ST | DT |
| rs543363163:G>A | Gly138Ser | 1000G | N | N | N | N | N | N | N | N | N | N | U | N | N | N | DT | DT |
| rs267606664:G>A | Gly145Asp | Cluster | N | N | D | N | D | N | D | N | D | N | N | D | D | D | DT | ST |
| rs531939919:C>T | Arg152Trp | 1000G | D | D | D | D | D | D | D | D | D | D | P | D | D | D | ST | ST |
| rs769455:C>T | Arg163Cys | 1000G, cluster, freq. | D | D | D | D | D | D | D | N | D | N | N | D | D | N | ST | DT |
| rs121918397:G>A | Arg163His | Cluster | N | D | D | D | D | N | N | N | D | N | N | N | D | N | DT | DT |
| rs121918394:A>C | Lys164Gln | Cluster | D | D | D | U | D | D | D | N | D | N | N | D | D | D | ST | ST |
| **rs7412:C>T** | **Arg176Cys** | 1000G, cluster, freq. | D | D | D | D | D | D | D | D | D | D | N | D | D | N | DT | DT |
| rs547472686:G>A | Ala225Thr | 1000G | N | N | D | N | D | N | N | D | D | N | U | N | D | D | DT | DT |
| rs567353589:G>A | Glu230Lys | 1000G | D | N | N | N | D | N | N | D | D | N | U | N | N | N | DT | DT |
| rs554251788:C>G | Arg233Gly | 1000G | N | D | D | N | D | N | D | D | D | N | U | D | D | D | ST | DT |
| rs530010303:C>T | Arg235Trp | 1000G | D | D | D | D | D | N | D | N | D | D | U | D | D | D | DT | DT |
| rs267606663:G>A | Arg242Gln | Cluster | D | N | N | N | N | N | D | D | D | N | N | D | D | N | DT | ST |
| rs121918395:C>T | Arg246Cys | Cluster | D | D | D | D | D | D | D | D | N | N | N | D | D | N | DT | DT |
| rs140808909:G>A | Glu262Lys | 1000G, cluster | N | N | D | N | D | N | D | N | D | N | N | D | D | N | ST | ST |
| rs190853081:G>A | Glu263Lys | 1000G, cluster | N | N | D | N | D | N | D | D | D | N | U | D | D | D | ST | DT |
| **rs557715042:G>T** | **Trp294Cys** | 1000G, freq. | D | D | D | U | D | D | D | N | D | N | P | D | D | D | DT | DT |

Convergent deleterious SNPs are in bold face.

^a^APOE amino acid positions is relative to GenBank Accession number NP_000032.1.

^b^1000G: SNP has been sequenced in the 1000 Genomes Project; freq.: Validated by frequency or genotype data: minor alleles observed in at least two chromosomes; cluster: Validated by multiple, independent submissions to the refSNP cluster

^c^N: Neutral; D: Deleterious; ST: Stabilizing; DT: Destabilizing; P: Pathogenic; U: Unknown.

**Table S3. Summary of structural validation parameters.**

| **Mutation** | **DOPE Score** | **Z-Score (Prosa II)** | **RamachandramPlot (%)** | | |
| --- | --- | --- | --- | --- | --- |
|  |  |  | | **Most favored Regions** | **Additional allowed regions** |
| Wild Type | -28406.160 | -4.01 | 90.9% | | 6.4% |
| Pro102Arg | -28437.195 | -3.70 | 88.9% | | 8.8% |
| Arg136Ser | -28319.149 | -3.85 | 89.9% | | 8.1% |
| Arg176Cys | -28368.377 | -3.92 | 90.2% | | 7.4% |
| Trp294Cys | -28237.611 | -3.79 | 89.6% | | 8.1% |

**Table S4. Average number of hydrogen bonds between known structural groups of ApoE3.**

| **Wild type** | **Loop N** | **Hinge H1** | **Hinge H2** | **Loop (218-227)** | **Helix C1** | **Loop (242-253)** | **Helix C2** | **Helix C3** | **Loop C** |
| --- | --- | --- | --- | --- | --- | --- | --- | --- | --- |
| **Loop N** | - | 0,000 | 0,000 | 0,000 | 0,000 | 0,000 | 2,401 | 2,401 | 0,417 |
| **Helix N1** | 0,000 | 0,000 | 0,000 | 0,000 | 0,000 | 0,000 | 0,409 | 0,096 | 0,002 |
| **Helix N2** | 0,000 | 0,000 | 0,000 | 0,000 | 0,000 | 0,000 | 1,539 | 0,000 | 0,008 |
| **Helix 1** | 0,000 | 0,000 | 0,000 | 2,041 | 0,004 | 0,000 | 0,000 | 0,000 | 0,006 |
| **Helix 1'** | 0,000 | 0,000 | 0,389 | 5,027 | 0,167 | 0,000 | 0,000 | 0,000 | 0,003 |
| **Helix 2** | 0,000 | 0,818 | 0,057 | 1,294 | 0,000 | 0,000 | 0,000 | 0,000 | 0,015 |
| **Loop (98-106)** | 0,000 | 0,181 | 0,000 | 0,000 | 0,000 | 0,000 | 1,673 | 0,000 | 0,006 |
| **Helix 3** | 0,000 | 1,933 | 3,295 | 0,000 | 0,000 | 1,537 | 4,074 | 0,001 | 0,039 |
| **Loop (144-148)** | 0,000 | 0,000 | 0,000 | 0,000 | 0,088 | 0,116 | 0,000 | 0,000 | 0,003 |
| **Helix 4** | 1,561 | 0,000 | 0,000 | 0,022 | 2,286 | 0,563 | 2,350 | 0,665 | 0,603 |
| **Pro102Arg** | **Loop N** | **Hinge H1** | **Hinge H2** | **Loop (218-227)** | **Helix C1** | **Loop (242-253)** | **Helix C2** | **Helix C3** | **Loop C** |
| **Loop N** | - | 0,000 | 0,000 | 0,000 | 0,749 | 0,000 | 1,956 | 1,956 | 0,460 |
| **Helix N1** | 0,325 | 0,000 | 0,000 | 0,000 | 0,000 | 0,000 | 3,852 | 3,852 | 0,002 |
| **Helix N2** | 0,000 | 0,000 | 0,000 | 0,000 | 0,000 | 0,000 | 1,486 | 0,443 | 0,010 |
| **Helix 1** | 0,000 | 0,000 | 0,000 | 0,000 | 0,314 | 0,000 | 0,000 | 0,000 | 0,009 |
| **Helix 1'** | 0,000 | 0,000 | 3,401 | 2,442 | 0,000 | 0,000 | 0,000 | 0,000 | 0,009 |
| **Helix 2** | 0,000 | 0,822 | 0,848 | 0,037 | 0,000 | 0,000 | 0,000 | 0,000 | 0,008 |
| **Loop (98-106)** | 0,000 | 0,049 | 0,000 | 0,000 | 0,000 | 0,000 | 0,028 | 0,000 | 0,006 |
| **Helix 3** | 0,000 | 6,739 | 0,989 | 0,000 | 0,000 | 5,937 | 5,036 | 0,000 | 0,092 |
| **Loop (144-148)** | 0,000 | 0,000 | 0,092 | 0,000 | 0,924 | 0,000 | 0,000 | 0,000 | 0,006 |
| **Helix 4** | 0,781 | 0,000 | 0,000 | 0,000 | 2,606 | 2,599 | 4,585 | 0,528 | 1,643 |
| **Arg132Ser** | **Loop N** | **Hinge H1** | **Hinge H2** | **Loop (218-227)** | **Helix C1** | **Loop (242-253)** | **Helix C2** | **Helix C3** | **Loop C** |
| **Loop N** | - | 0,000 | 0,000 | 0,000 | 0,391 | 0,000 | 0,744 | 0,744 | 0,694 |
| **Helix N1** | 2,415 | 0,000 | 0,000 | 0,000 | 0,023 | 0,000 | 0,381 | 0,381 | 0,000 |
| **Helix N2** | 0,000 | 0,000 | 0,000 | 0,000 | 0,000 | 0,000 | 0,097 | 0,086 | 0,019 |
| **Helix 1** | 0,000 | 0,000 | 0,000 | 0,004 | 0,444 | 0,000 | 0,000 | 0,000 | 0,004 |
| **Helix 1'** | 0,000 | 0,000 | 3,671 | 1,206 | 0,000 | 0,000 | 0,000 | 0,000 | 0,000 |
| **Helix 2** | 0,000 | 1,319 | 0,055 | 0,566 | 0,000 | 0,000 | 0,000 | 0,000 | 0,018 |
| **Loop (98-106)** | 0,000 | 1,520 | 0,000 | 0,000 | 0,000 | 0,000 | 1,894 | 0,000 | 0,018 |
| **Helix 3** | 0,000 | 5,314 | 1,076 | 0,000 | 0,000 | 1,385 | 3,603 | 0,000 | 0,019 |
| **Loop (144-148)** | 0,000 | 0,000 | 0,000 | 0,000 | 0,013 | 0,833 | 0,000 | 0,000 | 0,009 |
| **Helix 4** | 0,264 | 0,000 | 0,000 | 0,000 | 2,648 | 2,276 | 6,165 | 1,426 | 0,037 |
| **Arg176Cys** | **Loop N** | **Hinge H1** | **Hinge H2** | **Loop (218-227)** | **Helix C1** | **Loop (242-253)** | **Helix C2** | **Helix C3** | **Loop C** |
| **Loop N** | - | 0,000 | 0,000 | 0,000 | 2,499 | 0,000 | 0,345 | 0,340 | 0,016 |
| **Helix N1** | 0,982 | 0,000 | 0,000 | 0,000 | 0,000 | 0,000 | 1,363 | 1,357 | 0,008 |
| **Helix N2** | 0,000 | 0,000 | 0,000 | 0,000 | 0,000 | 0,000 | 1,241 | 0,000 | 0,013 |
| **Helix 1** | 0,022 | 0,000 | 0,000 | 0,118 | 0,211 | 0,000 | 0,000 | 0,000 | 0,010 |
| **Helix 1'** | 0,000 | 0,000 | 0,605 | 3,147 | 0,264 | 0,000 | 0,000 | 0,000 | 0,003 |
| **Helix 2** | 0,000 | 1,469 | 0,057 | 1,126 | 0,000 | 0,000 | 0,000 | 0,000 | 0,033 |
| **Loop (98-106)** | 0,000 | 0,546 | 0,000 | 0,000 | 0,000 | 0,000 | 2,156 | 0,000 | 0,011 |
| **Helix 3** | 0,000 | 2,245 | 0,003 | 0,000 | 0,000 | 3,074 | 3,733 | 0,000 | 0,014 |
| **Loop (144-148)** | 0,000 | 0,000 | 0,000 | 0,000 | 0,064 | 0,000 | 0,000 | 0,000 | 0,002 |
| **Helix 4** | 0,183 | 0,000 | 0,000 | 0,003 | 1,865 | 1,088 | 7,450 | 3,398 | 0,241 |
| **Trp294Cys** | **Loop N** | **Hinge H1** | **Hinge H2** | **Loop (218-227)** | **Helix C1** | **Loop (242-253)** | **Helix C2** | **Helix C3** | **Loop C** |
| **Loop N** | - | 0,000 | 0,000 | 0,000 | 1,083 | 0,000 | 1,512 | 1,512 | 0,958 |
| **Helix N1** | 1,733 | 0,000 | 0,000 | 0,000 | 0,000 | 0,000 | 0,516 | 0,516 | 0,001 |
| **Helix N2** | 0,000 | 0,000 | 0,000 | 0,000 | 0,000 | 0,000 | 0,002 | 0,000 | 0,007 |
| **Helix 1** | 0,129 | 0,000 | 0,000 | 0,645 | 1,118 | 0,000 | 0,000 | 0,000 | 0,013 |
| **Helix 1'** | 0,000 | 0,000 | 0,475 | 1,091 | 0,000 | 0,000 | 0,000 | 0,000 | 0,036 |
| **Helix 2** | 0,000 | 1,431 | 0,935 | 0,022 | 0,000 | 0,000 | 0,000 | 0,000 | 0,017 |
| **Loop (98-106)** | 0,000 | 1,447 | 0,000 | 0,000 | 0,000 | 0,000 | 0,826 | 0,000 | 0,007 |
| **Helix 3** | 0,000 | 5,656 | 0,331 | 0,000 | 0,000 | 2,525 | 4,451 | 0,000 | 0,018 |
| **Loop (144-148)** | 0,000 | 0,000 | 0,000 | 0,000 | 0,224 | 0,172 | 0,000 | 0,000 | 0,130 |
| **Helix 4** | 0,040 | 0,000 | 0,000 | 0,000 | 1,953 | 0,411 | 2,840 | 1,040 | 1,788 |

The groups are named according to Chen et al., 2012 9. In the case of loops without a specific denomination of numbers corresponds to the number of residues (e.g., Loop 98-106 represents the loop between ApoE residues 98 and 106).


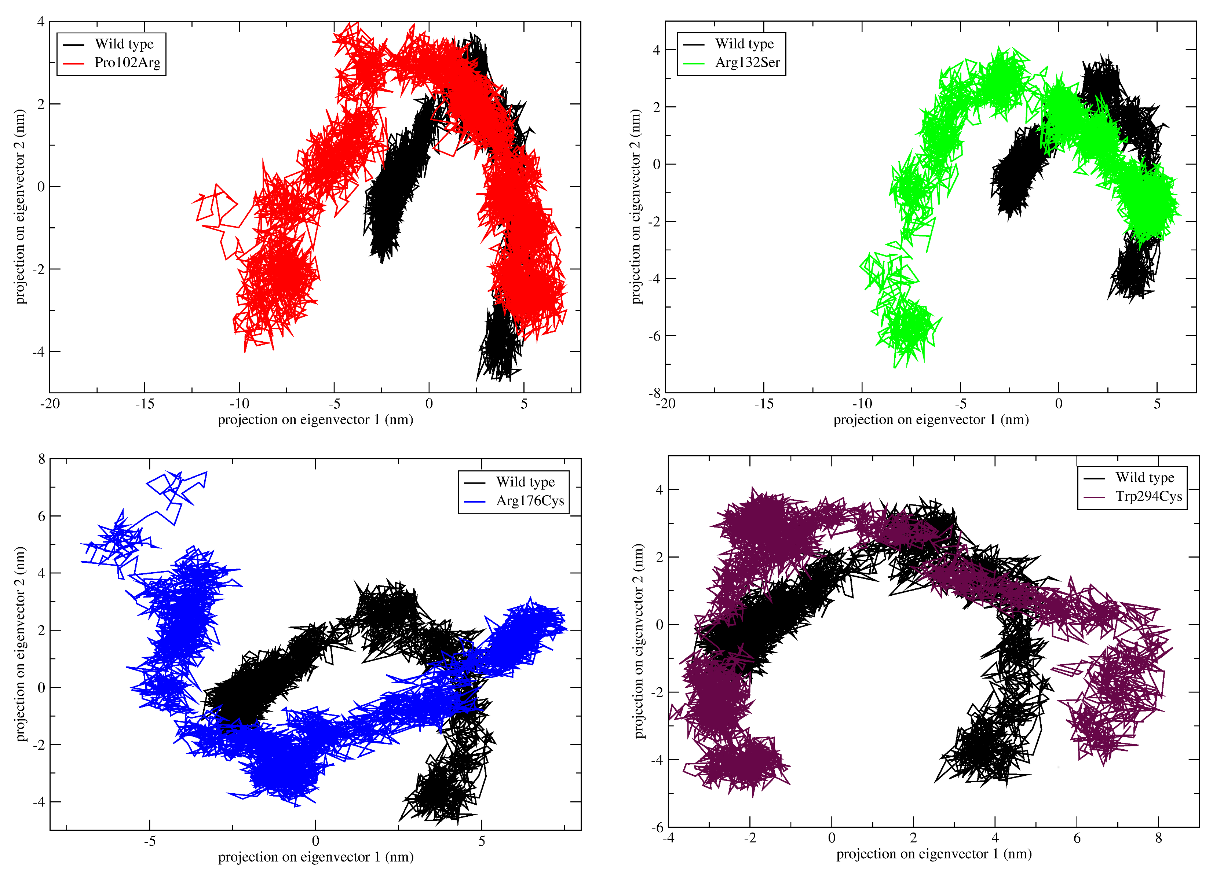


**Figure S1. Covariance matrices of the variants and wild type apoE in phase space along the first two principal eigenvectors.** Most of the variants shown lower traces of diagonalized covariance matrix than wild type excepting the Arg132Ser variant. The trace of diagonalized covariance matrix (nm²) of the variants are: Pro102Arg (48.0029), Arg132Ser (42.0822), Arg176Cys (40.1135) and Trp294Cys (39.6023). Whereas wild type’s trace of diagonalized matrix is 24.5582. The variants are identified in the plots by colors.


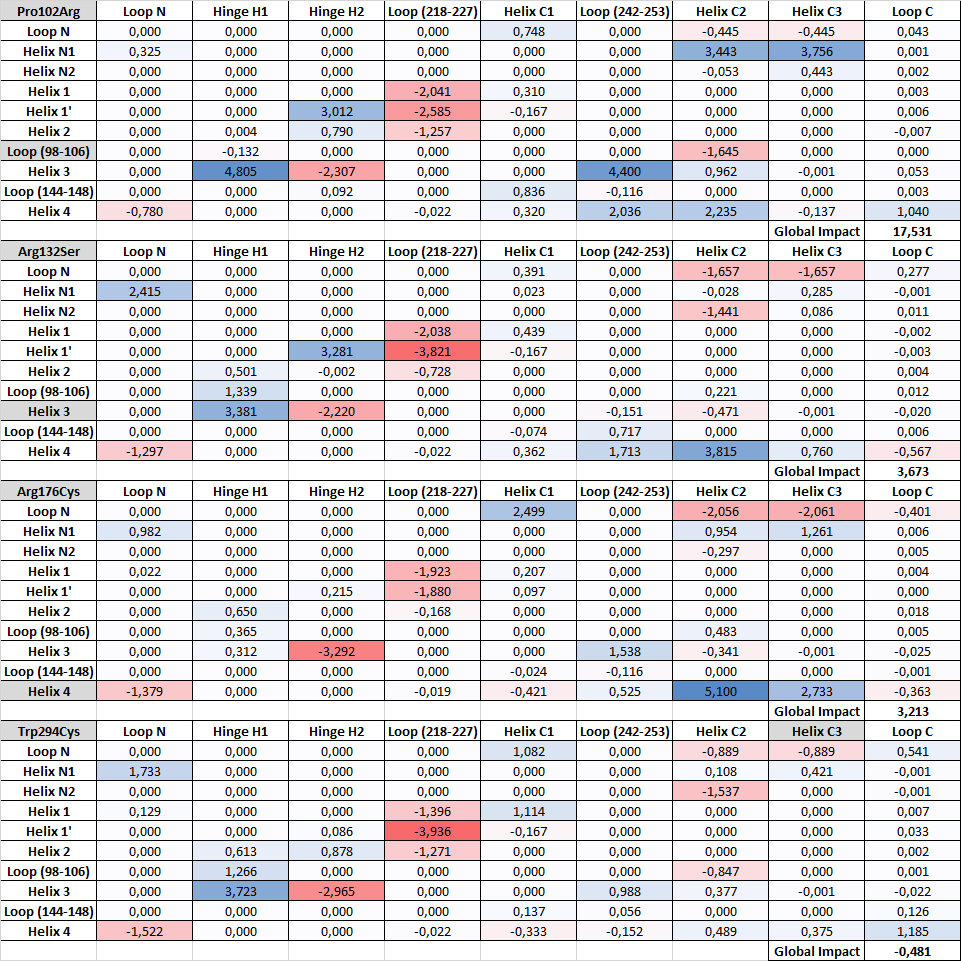


**Figure S2. Heatmap showing the difference between known structural group interactions in wild type and variants' simulations.** The calculated numbers refer to comparison of the variants with respect to the wild. Regions where there is average loss of interactions are marked in red tones and regions with gain of interactions are marked in shades of blue. The global impact refers to sum of all gains and loss of interactions. Most of the variants showed loss of interactions except Trp294Cys where there was overall overall gain above 7. The groups are named according to Chen et al., 2012 9. In the case of loops without a specific denomination of numbers corresponds to the number of residues (e.g., Loop 98-106 represents the loop between ApoE residues 98 and 106). Regions marked in gray refer to the position of the mutation.
